# High-energy ion beams generated with high efficiency using laser-driven 3D microstructures

**DOI:** 10.1038/s41598-025-21798-6

**Published:** 2025-10-29

**Authors:** Sergei Tochitsky, Nuno Lemos, Raspberry Simpson, Elizabeth Grace, Arthur Pak, Tammy Ma, Joshua Luoma, Frederico Fiuza, Dan Haberberger, Alex Haid, Katharine Knolker, Chan Joshi

**Affiliations:** 1 Department of Electrical Engineering, University of California Los Angeles, Los Angeles, CA 90095 USA; 2 Lawrence Livermore National Laboratory, Livermore, CA 94550 USA; 3 GAP/Instituto de Plasmas e Fusão Nuclear , Instituto Superior Technico, Lisbon, 1049-001 Portugal; 4 Laboratory for Laser Energetics, Rochester, NY 14623 USA; 5 General Atomics, San Diego, CA 92121 USA

**Keywords:** Physics, Plasma physics, Plasma-based accelerators

## Abstract

Laser-driven ion acceleration in plasma is being proposed as a source of ion beams with a high peak current that can be useful in many fields of science and medicine. Using this method, high proton energies have been achieved by increasing the laser power and by using ultrathin (≤ 200 nm) foils. However, this approach is limited by survivability of the nanotargets to laser prepulses and by difficulty in controlling the plasma acceleration properties. Here, we introduce a new target platform using two-photon polymerization, 3D laser-printed “clone” microstructures with average densities lower than solid that are relatively insensitive to the laser prepulse. Two types of microstructured targets consisting of either a multilayered log-pile or a stochastic arrangement of one micron diameter wires are used. Both demonstrate a higher energy and higher yield proton acceleration compared to thin solid-density foil targets by the robust target normal sheath acceleration (TNSA) mechanism. We find that when such 10–20 μm thick structures are irradiated with a petawatt laser, protons with energies up to 110 MeV and a laser-to-proton conversion efficiency of ~ 10% are obtained. Our work suggests that such microstructures optimized for 60–200 MeV compact proton accelerators are promising for future radiotherapy and other applications.

 The advent of very high-power PW-class 1 μm lasers has enabled experiments on relativistic laser-plasma interactions in which a significant fraction of laser pulse energy can be transferred to high energy plasma electrons which is subsequently transferred to ions^1,2^. The unique properties of laser-accelerated ion beams have been recognized and exploited in radiography of high-energy density settings for probing highly transient electrical and magnetic fields in plasmas^3,4^, radiation-induced processes in solid targets^5^, isochoric heating of matter^6^ and developing a new approach to radiobiology based on extremely high single-shot dose rates^7–9^. The latter application may have a significant societal impact because the current approach to ion-based radiotherapy relies on very bulky and expensive radio-frequency (RF) accelerators and beam delivery gantries. Several decades of clinical proton oncology has established energy requirements of proton beams as being ~ 60 MeV for readily accessible tumors e.g. eye and ≥ 200 MeV for deep seated tumors, with a typical dose rate of about 20 Gy/min over ~ 5 minutes^10^. In principle, far higher dose rates, delivered in a much shorter time duration in the range 40–300 Gy/s, are being considered because of their potential to generate the FLASH effect^11–13^. Such ultra-high dose rate seems to lead to relative protection of the normal tissue that is exposed to the FLASH ion beam as compared with a conventional dose. Laser-accelerated proton/ion sources are very promising since they can deliver a large number of particles per shot (≥ 10e10or a peak current > 100 A), tightly confined in time (~ 1–10 ps) that can be focused to a small spot size(~ 10 μm)^14^. Therefore, dose rates for either H or C ions from the same laser source can be several orders of magnitude above what state-of-the-art RF accelerators produce. There are several techniques for selecting a relatively narrow energy slice from the laser-generated wide energy spread ion spectra^15–17^, but the required high charge, and energy (60–200 MeV for H^+^) in a controllable and reproducible manner remains an unsolved challenge. Reproducibility of high-energy of proton beams is of particular importance while probing the dynamics of electric and magnetic fields in high-energy density laboratory plasmas typically driven by single-shot kJ-class picosecond pulsed laser systems.

When a focused PW power laser beam interacts with a few micron thick solid foil in vacuum, the laser instantly ionizes the front surface of the target. This plasma absorbs part of the laser energy and during this interaction energetic electrons are accelerated by the laser field to energies characterized by a hot electron temperature, T_hot_≥ 1 MeV. These electrons are initially directed inwards eventually reaching the rear side. At the rear surface, the accelerating field is set up by the expulsion of a hot electron cloud into vacuum producing a negatively charged sheath field that is several Debye lengths thick, normal to the surface. The typical electrostatic field strength of this capacitor-like configuration can be several TeV/m. It is this field that accelerates ions which is why this mechanism is known as the target normal sheath acceleration (TNSA) mechanism^2,18^. Theoretical model suggests that the maximum kinetic energy of protons E_max_ scales roughly as T_hot_^2,18,19^. This scaling has stimulated research on ways of enhancing electron heating and maintaining the source of hot electrons for possibly several picoseconds without ramping up the laser power. Towards this goal a low-density foam or microwire/nanowire arrays were placed in front of the micron size foil^20–24^ or a layer of a low-density plasma was generated using a laser prepulse^25^. Both approaches allowed for additional electron heating in an under critical density plasma. Note, that the critical plasma density, n_c_ equals to 1 × 10^21^ cm^−3^ for a laser wavelength of 1 μm.

In the last two decades, several variations of ultrathin ~ 100–200 nm thick nanofoils were explored. Here, for a sufficiently high laser intensity, radiation pressure can push all electrons in the focal volume forward and ions follow forming a moving double-layer (light-sail radiation pressure acceleration^2,26,27^. Alternatively, the plasma can be made relativistically transparent to the laser pulse, enhancing the T_hot_ value^28–31^. It should be noted that currently there are two main avenues in laser-driven ion acceleration research using PW class laser-drivers: picosecond 0.1–1.1 kJ glass-based sources operating in a single-shot regime and femtosecond lower-energy but higher repetition rate Ti: sapphire based systems. The latter approach recently demonstrated remarkable 150 MeV protons from nanofoils generated in the relativistic transparency regime^32^. Unfortunately, the survival of such ultrathin targets irradiated by realistic PW-power, laser pulses is jeopardized by the presence of a picosecond/nanosecond prepulse and/or onset of a transverse plasma instability^33^.

To develop a robust platform for ion acceleration in PW laser interactions with solid targets, we explore a novel reproducible target design, two-photon polymerization (2PP) 3D laser-printed multilayer structures. They increase both the laser absorption - to enhance the ion yield - and the temperature of the hot electrons that increases the maximum ion energy. We have produced low-average density, relatively thick (~ 10–50λ, where λ is the laser wavelength) both periodic Log-Pile (LP) and non-periodic STochastic (ST) wire microstructures and demonstrated acceleration of up to ~ 110 MeV H^+^ and > 330 MeV C^6+^ ions with a > 10X increased particle yield in comparison with 2–5 μm CH foils. Moreover, we show control of the cut-off ion energy in the range of 80–110 MeV by tailoring the structure’s density and length. We have found that usage of ≥ 10 μm free-standing multilayer wire-structures decouples the rear surface TNSA from the laser prepulse effects thus facilitating reproducible particle acceleration.

## Results

**Fig. 1:**
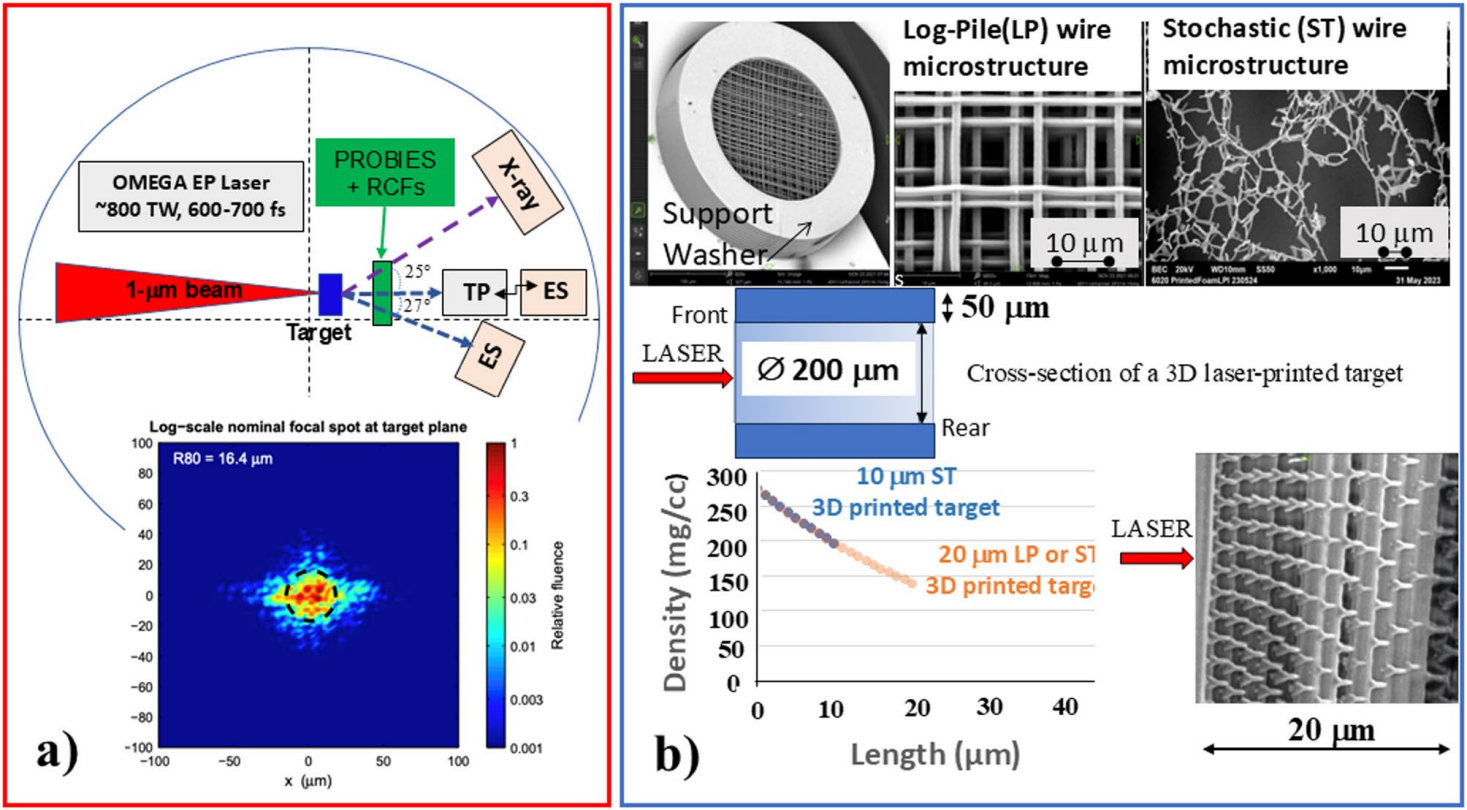
Experimental set-up and description of two-photon polymerization 3D printed targets. **(a)** Simplified sideview of the experimental set-up. One micron laser pulses are focused by an ~ *f*/2 off-axis parabola onto a target at normal incidence. The generated forward propagating particle and x-ray beams were analyzed by a Thomson Parabola (TP) spectrometer or an Electron Spectrometer (ES) on-axis, and by ES or X-ray spectrometer placed, respectively at 27° and 25° to the laser propagation direction. The spatial and energy characteristics of the proton beams were simultaneously measured by a Proton Beam Imaging Energy Spectrometer (PROBIES) equipped with a stack of radiochromic films positioned ~ 5 cm behind the target. The inset represents a fluence map of typical laser beam in the focal plane with a peak a_0_ ≥ 20 dashed black circle has a radius of 16.4 μm and contains > 80% of the total energy. **(b)** Overall dimensions and density profile of 2PP 3D laser-printed microstructures used in the experiments. Scanning electron microscope images of a Log-Pile (LP) 3D microstructure printed in a ∅200 μm support washer and zoomed-in SEM images of LP and STochastic (ST) wire microstructures taken at the rear side of a 20 μm thick target. An SEM top view of a washer-free metrology 20 μm thick LP microstructure indicating high-density side irradiated by laser. The consecutive layers of laser printed LP structure have a lateral shift.

The experiments on laser-driven ion acceleration were conducted using the OMEGA EP ~ 0.8 PW (≤ 500 J in ~ 600 fs per pulse) glass laser system at the Laboratory for Laser Energetics, for which an average peak intensity in a focused beam reached ≥ 5 × 10^20^ W/cm^2^ with the normalized vector potential, a_0_ = eA/m_e_c^2^≈20. For the focused laser beam, the power contrast is about 10^9^, the intensity contrast is about a factor of 10 better because the amplified spontaneous emission as shown by detailed measurements in the OMEGA EP laser system could not be focused as well. The cross-correlation measurements have shown that the prepulse is mainly developed in the − 100 ps to 0 ps time window and contrast degradation to < 10^6^ on a few ps scale is attributed to high-frequency spectral phase noise in the grating stretcher and compressor^34^. As described in the Methods and shown in Fig. 1a, for each shot the generated ion/proton beams as well as electron and X-ray emission were detected independently on-axis by using a radiochromic film (RCF) stack with a 15 mm hole and a Thomson parabola (TP) as well as by electron (ES)/X-ray spectrometers at different angles to the laser axis, respectively.

Ion acceleration from 2PP 3D LP and ST wire structures were investigated. The LP/ST microwire structures (shown in Fig. 1b) are 3D laser-printed by General Atomics using the 2PP nanoprinting technique. This 3D lithography technology allows for fabrication of ~ 0.5–1 μm diameter dielectric wire structures that can be made identical, designed for an arbitrary averaged material density profile and with/without regular organization of microwires^35–38^. The microwires are made of a polymer C_55_H_66_O_25_ with a mass density of 1.18 g/cm^3^ and were printed inside of a 200 μm diameter solid washer that supports the structure and is used for mounting a stalk. In our experiment, the individual wire diameter was ≤ 1 μm and the target’s average neutral density was controlled by changing the period (void) size (see Methods). An example of the target with a graded density is shown in Fig. 1b. Here, for a 20 μm long target, the spacing between wires varied from ~ 2.5 μm at the front to ~ 10 μm at the exit as seen in SEM images of the exit side of the target. We also show the top view SEM image of metrology (without washer) structure clarifying the laser direction and lateral offset for consecutive wire layers in the target. Such ~ 2λ spacing between wires at the front of the target is close to the optimum for maximizing the laser absorption and proton acceleration as was shown for both periodic nano/micro wire-arrays pointed towards the laser^22,39,40^ and nonperiodic cones or nanoholes^41^. Note, that a target with 275 mg/cm^−3^ neutral density, if fully ionized corresponded to the peak plasma density ≥ 19n_c_, which is still ~ one fifth of the solid density of CH foils.

**Fig. 2:**
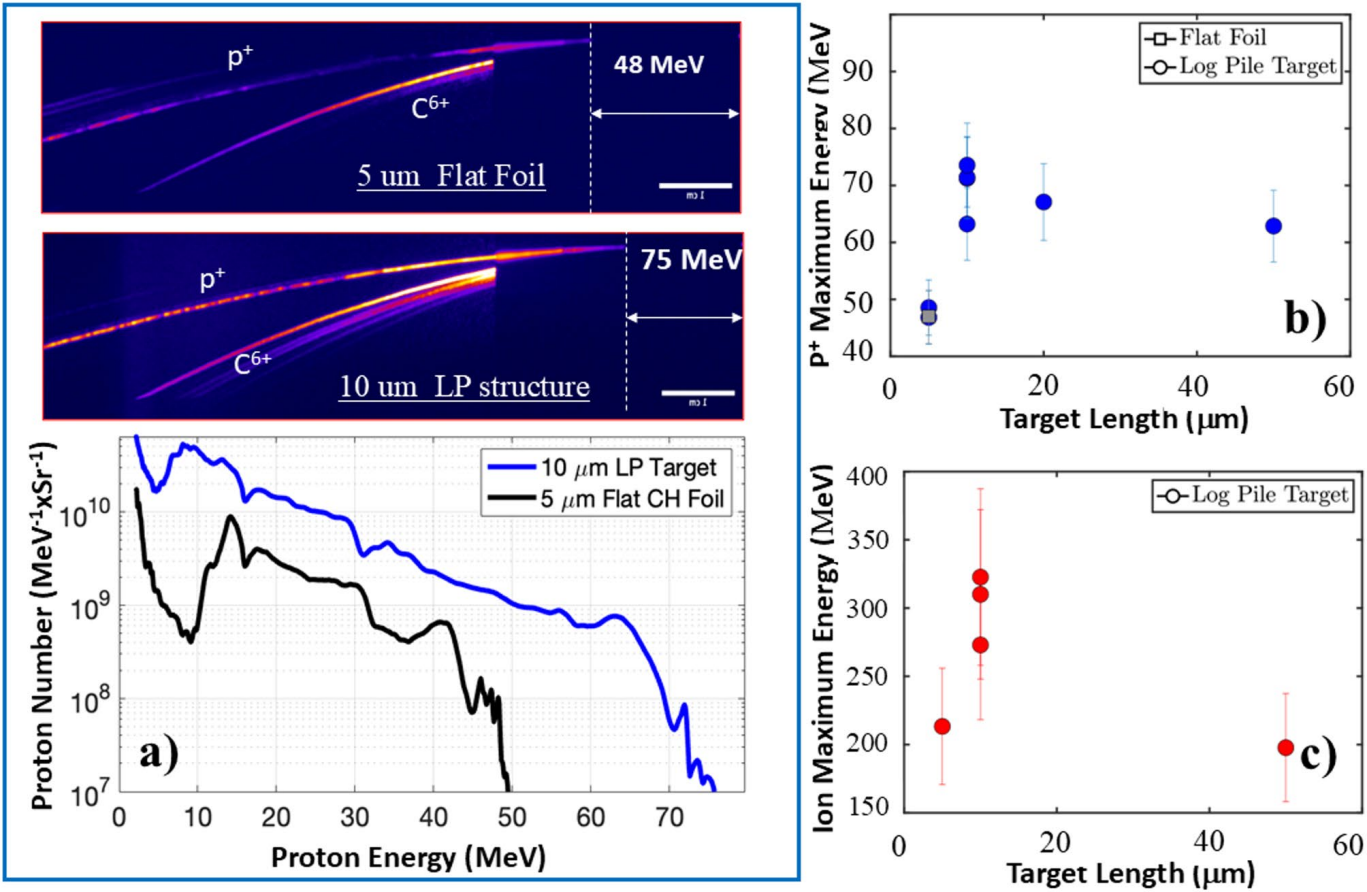
Thomson Parabola spectrometer data of proton/ion acceleration in 2PP 3D laser-printed microstructures. **(a)** An example of TP data taken for a 10 μm thick LP microstructure and a 5 μm thick CH flat foil. Besides the protons, heavier ions are detected. The majority of accelerated ions are C^6+^ with smaller amounts of O^8+^. In order to differentiate between proton and overlapping heavier C^6+^+ O^8+^ traces, a 1 mm thick Al plate-filter is installed in front of the part of read-out image plates of the spectrometer blocking the high-energy part of heavier ion spectrum. Accelerated proton spectra for a LP target and a 5 μm flat foil for comparison indicate significant enhancement of both proton yield and the cut-off energy in the case of a 3D microstructured wire target. **(b)** Maximum proton energy as a function of the target length for LP microstructures. A grey square data point corresponds to the 5 μm CH flat foil. **(c)** Maximum C^6+^ ion energy as a function of the LP target length indicating that the highest energy ions and protons are observed for the same 10 μm target length. Thereafter, both ion and proton energies decrease slowly in 2b and 2c.

We observed a clear advantage of microstructed targets compared to a flat foil for generation of high-energy protons in the forward direction. In Fig. 2a we show an example TP spectrum obtained both for a 10 μm thick LP structure and 5 μm thick flat CH foil and extracted proton spectra. The proton’s cut-off energy for the LP target reached ~ 75 MeV against ~ 48 MeV for the CH foil and the proton yield was more than 5X larger for the LP target than that for a foil in the entire 15–50 MeV range of comparison.

Data in Fig. 2a also indicates that a 3D printed structure is much more efficient in converting light into protons and simultaneously significantly increases the cut-off proton energy. The efficiency is presumably related to higher laser absorption of photons trapped in the voids of the targets while higher maximum proton energy is the result of a higher T_hot_. A laser-to-proton conversion efficiency in the 10–55 MeV range was estimated to be around 8% for the 10 μm LP structure as opposed to 1.4% for the foil. The net conversion efficiency for the LP microstructure exceeded ~ 10%. Note, that in a recent study of TNSA enhanced by microtube/micropillar arrays printed on the top of a 1-µm Co substrate, a significant laser-to-ion conversion efficiency was reported but without increase in the maximum proton/carbon energy^24^.

For the same 10 μm thick LP targets, we have also tested sensitivity of microstructures to the finite laser contrast by comparing ion spectra produced from targets with and without a 1 μm protective plastic layer printed in the front. Maximum energy in both cases was the same that allows us to deduce that plasma or shock formation on the front surface of the microstructure by the prepulse does not affect sheath acceleration in the plasma-vacuum interface at the rear. This is different from extreme sensitivity to the laser contrast reported for ultrathin foils^28–32^. Note, that distributed absorption of laser into the discrete multilayer wire structures is inefficient to the shock formation/propagation in the plasma- the main mechanism behind deformation and possible degradation of ion density gradient at the rear of the target^42,43^.

**Fig. 3:**
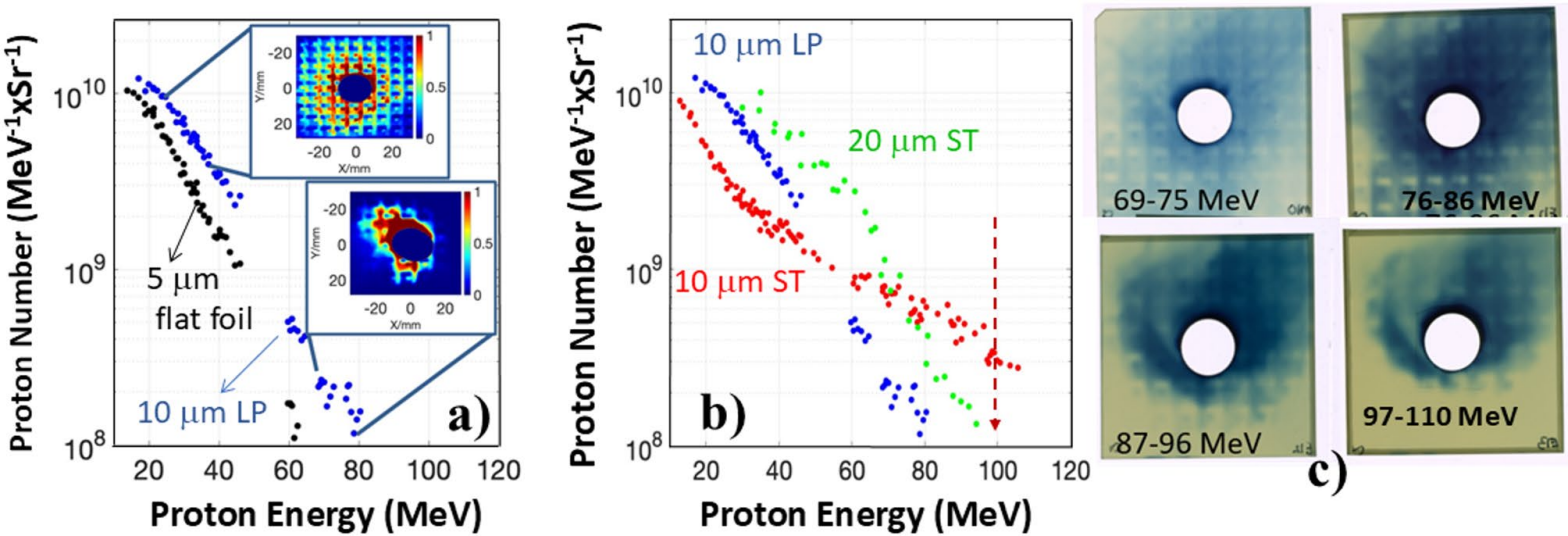
Proton spectra generated in log-pile and stochastic wire microstructures measured by PROBIES. **(a)** Comparison of the measured proton spectra for a 10 μm LP wire structure and the 5 μm flat CH foil. The insets show examples of the proton beam profiles recorded 50 mm in the forward direction by RCFs for different proton energy ranges 24–34 MeV and 76–86 MeV. Smaller beam size for higher energies implies decreasing divergence with increasing energy as expected from the TNSA mechanism. The small square structure on RCFs called “city block” structure is imprinted on a proton beam by a multi-step Al plate-filter placed in front of the film stack. **(b)** Proton spectra for different types of microstructured targets 10 μm LP, 10 μm ST and 20 μm ST wire structures. The proton number at the maximum cut-off energy of ~ 110 MeV exceeds a 100 MeV level indicated by a dashed red arrow was still above the noise level of RCFs. The detection threshold was 8 × 10^7^ protons/MeVxSr. **(c)** Example proton beam distribution from a 10 μm LP structure, as measured by the RCF detectors covering the energy ranges of 69–75 MeV (MD-V3 film), and 76–86 MeV, 87–96 MeV, and 97–110 MeV (all EBT-3 film).

To optimize the maximum energy of protons, we varied thickness of LP targets from 5 to 50 μm while keeping a self-similar density profile. In Fig. 2b we present the results of TP measurements of the proton cut-off energy as a function of target length. The maximum energy was reached in a 10 μm thick microstructure. In Fig. 2c, we show the maximum observed energy of the heavier ion species C^6+^ and O^8+^ assuming that in the integrated signal most of the contribution to this TP curve is from the C^6+^ ions. Note, that for the 2PP polymer material of wires the initial contamination of carbon is more than two times higher than that of oxygen atoms. The maximum ion energy of ≥ 330 MeV was recorded for 10 μm thick LP targets and the total number of measured C^6+^ ions was about 2.3X larger than that generated in a flat 5 $\:\mu\:$m thick foil. All recorded proton and ion spectra for the ≥ 10 μm LP targets have had monotonically decreasing number of particles within beams emitted in a forward ± 25° cone normal to the rear surface suggesting that the layers of wires at the rear remained at the solid-density long enough to facilitate the efficient TNSA. To summarize, for 5 μm thick LP structures, not only the maximum proton energy was low, but the beam profile was very different from that presented in Fig. 3. The protons were steered off the laser axis and a spatially fractured proton beam was recorded indicating distortion of the 2D sheath field at the rear surface of the target. This observation was additionally supported by shots with half of the laser power when the proton beams of lower energies from the same 5 μm targets became round and homogeneous. For the longest 50 μm targets, in the experiment the proton energies despite somehow increased uncertainty of TP measurements were consistently lower than that for 10–20 μm thick LP wire structures.

The proton beams from microstructures were also measured independently for each shot by a proton spectrometer built using a 13 layered RCF stack that captured the forward accelerated beam of protons (see Fig. 1a), The film stack was preceded by either an Al or W plate of machined filters to increase the energy resolution of a PROton Beam Imager and Energy Spectrometer (PROBIES)^44^. Once again, the maximum energy protons were found be similar with both diagnostics. The maximum proton energy reached ~ 80 MeV for a 10 μm thick LP microstructure. In Fig. 3a we show examples of the proton beam profiles for different energy ranges. The “city block” square structure of the recorded profiles is due to PROBIES step-filters as detailed in Methods. Apparent decrease in the divergence angle for higher energy particles is a characteristic feature of TNSA^2,19^.

To check whether wire structure affects the laminarity of generated proton beams we also tested the LP structure of optimal length with a 2 μm thick solid layer printed at its rear surface. Within accuracy of measurements, 10 μm LP targets with or without the layer demonstrated very similar beam spatial and spectral parameters suggesting no measurable difference in homogeneity of the sheath field at the plasma-vacuum interface. This observation points to an existence of transversely homogeneous sheath field distribution for the microstructures that is likely due to plasma filling the spaces around the ionized wires at the time window when ion acceleration takes place.

We tested two types of printed targets: periodic LP and non-periodic ST wire microstructures. It is important that nanoprinting technology is capable of fabricating multiple “clones” of these 3D stochastic structures in Fig. 1b making the differences between the ST and LP structures reproducible. As shown in Fig. 3b, for the optimal 10 μm long ST target, proton energies around 110 MeV were measured versus 80 MeV for the LP target. This trend was observed for each target thickness. In Fig. 3c one can see a significant proton population detected by the last RCF film of the stack covering the energy range of 97–110 MeV (as well as detectors covering 69–96 MeV energies) with a clear imprint of square “city block” structure related to PROBIES. Note, that for the latter experiments the cut-off energy value was limited not by the noise level on the RCF film but by the detector’s energy range indicating that the actual maximum proton energy for ST target was likely even higher. It is important that 20 μm ST target produced less energetic proton beams up to 95 MeV. Thus, from experimental observations we may suggest that 2PP 3D laser-printed targets can be designed to give a specified energy of protons/ions depending on the application needs.

**Fig. 4:**
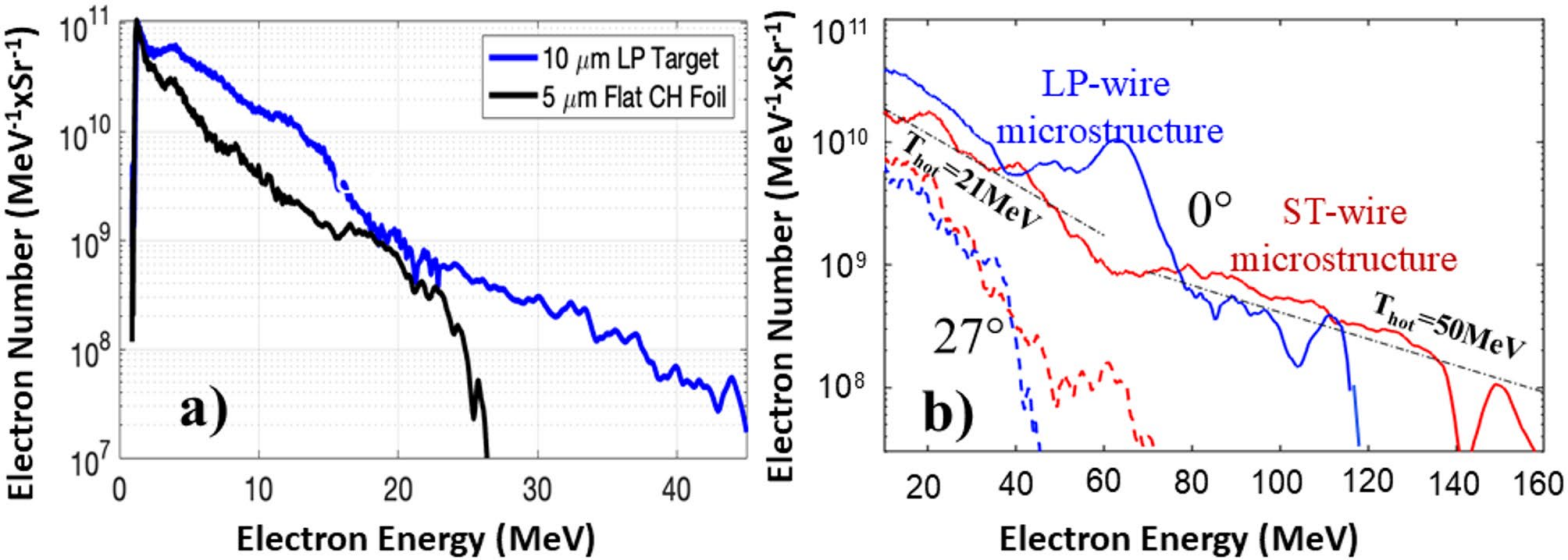
Experimentally measured spectra of electrons emitted on-axis and off-axis by the laser irradiated 3D printed microstructures. **(a)** Electron spectra recorded by an electron spectrometer positioned at 27° in respect to the laser beam axis for a 10 μm LP target (blue line) and a 5 μm flat foil (black line). **(b)** Electron spectra observed for LP (blue lines) and ST (red lines) wire microstructures measured on the laser axis at 0° (solid lines) and at 27°(dashed lines) for a target thickness of 10 μm. Two-temperature distribution of laser-heated electrons is recorded for a 3D stochastic microstructure: T_hot_~21 MeV for energies in the range of 20–60 MeV and super-thermal electrons with T_hot_≤50 MeV for high-energy tail 80–160 MeV electrons.

In TNSA the maximum ion energy depends on the magnitude of the sheath potential and its lifetime. Both quantities in turn depend on the fraction of the laser energy that is coupled to the plasma electrons and the laser pulse duration and peak intensity. The effective electron heating can be qualitatively inferred from measuring the spectrum and the yield of relativistic electrons that escape the target^45^. Using magnetic spectrometers, we measured electron emission spectra 27° off-axis for every target and on-axis for a few shots. In Fig. 4a from the off-axis spectrometer data, it is apparent that a 10 μm LP structure generated 5-10X more hot electrons in the range of 2–15 MeV than that in a flat foil target and the energetic tail extended to 45 MeV. Moreover, in a series of measurements of the electron spectra on-axis versus that off-axis we observed a clear signature of directionality of the accelerated hot electrons. In Fig. 4b we summarize the electron measurements for both LP and ST targets of optimal length. First, electrons measured at 0° are hotter (solid lines) than that at 27° (dashed lines). At lower energies < 50 MeV, the estimated T_hot_ increased from 14 MeV for off-axis (not shown in Fig. 4b) to T_hot_≥21 MeV for the on-axis electrons. Second, the on-axis electron spectra showed a two-temperature distribution with a super-thermal component reaching T_hot_~50 MeV. This high-energy tail extends to above 140 MeV for the ST and ≥ 100 MeV for the LP target of the same length. Using the same peak target density both for LP and ST targets along with the same wire diameter perhaps explains why the superponderomotive electron heating characterized by very high T_hot_~50 MeV for both of them is similar. However, higher electron energies on-axis for ST targets support the notion that more electrons are being heated to higher temperature in a stochastic microstructure consistent with the higher energy protons measured in Fig. 3b. Possible contribution of direct laser acceleration (DLA) of electrons as physical mechanism behind the observed enhanced electron acceleration will be discussed below. It is interesting, that bump observed in a blue solid curve in Fig. 4b may indicate efficient acceleration of a copious number of electrons with specific energies thus showing its potential for generation of narrow band X-rays. We also collected x-ray emission data that shows harder x-ray generation with these targets, indicative of high electron temperatures in the target, but unfortunately this diagnostic does not have the resolution and sensitivity to directly correlate the x-ray data with the observed details for the electron spectrum.

Additionally, we observed a clear correlation between very high T_hot_ in plasmas and high-energy X-ray emission measured at approximately 25° in the forward direction (see Fig. 1a). These X-ray spectra (measured from 20 KeV to 1 MeV) are better indicators of the hot electron temperature inside the target than the escaping hot electrons. The measurements demonstrated that the microstructures produced more high-energy X-rays in comparison with a flat foil despite the latter having a slightly greater areal density. It is interesting that the X-ray diagnostic showed a very clear correlation on the target’s length scaling producing significantly more high-energy X-rays for both LP and ST microstructures of the optimal length.

**Fig. 5:**
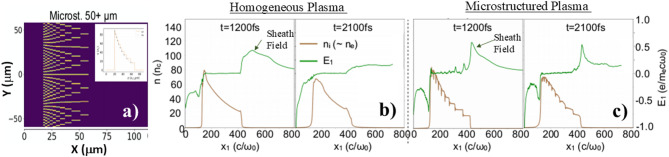
Comparison between homogeneous and microstructured wire plasma in 2D PIC simulations. **(a)** A cross-section of a simplified 50 μm log-pile wire microstructure used for this 2D PIC simulations. In simulations, the peak laser intensity corresponds to a_0_ = 20. **(b)**-**(c)** Snapshots of transversely averaged plasma density profile (left axis) and longitudinal electrical field (right axis) taken at 1.2 ps and 2.1 ps for a homogeneous plasma **(b)** and a microstructured wire related plasma **(c)** taken at t = 1.2 ps and t = 2.1 ps.

Previous experimental measurements using both femtosecond^39,40^ and picosecond^22^ pulses shown that due to the increased interaction between the laser and the structure’s surface more electrons are ejected from the wire boundaries mainly through Brunel-type and J × B absorption processes, which then can be further accelerated in the gaps and, therefore enhance the ion yield and energy manifold. Complicated interplay between different mechanisms of electron heating such as JxB^46^, and DLA^47–50^ and transport both in the bulk and boundary parts as well as dynamics of TNSA was studied by 2D particle-in-cell (PIC) simulations using code OSIRIS^51^[see the Methods]. As the first step, for the same averaged CH plasma density profile and the laser strength of a_0_ = 20 we compared a preformed (homogenized foam-like^50^ plasma with an LP microstructure simplified in the way shown in Fig. 5a. For this simulations, a 50 μm thick LP target with a tailored density profile made of a manifold of wires orthogonal to the cross-section plane mimicking those in 3D printed microstructures can be seen in Fig. 1b in the SEM top view image of a free-standing (without washer) laser-printed LP structure. As apparent in Fig. 5a, this microstructure also has a clear lateral offset of consecutive wire layers important for the light trapping in laser-plasma interactions.

The key physics finding from this comparative numerical analysis in Fig. 5b and c is that the presence of dense wires allows for sustaining higher sheath field magnitude over longer period of time in comparison with the homogeneous preformed plasma with the same tailored density profile. The lifetime of the sheath field of the order of 2–3 ps was facilitated by relatively large micrometer size CH wires. Moreover, the sheath field peaks at the plasma vacuum boundary where the most ion acceleration is anticipated according to the TNSA mechanism. The proton cut-off energy and the particle yield were substantially higher in the case of the microstructured plasma. Similar difference is anticipated if instead of the tailored homogeneous plasma a flat solid foil is considered.

**Fig. 6:**
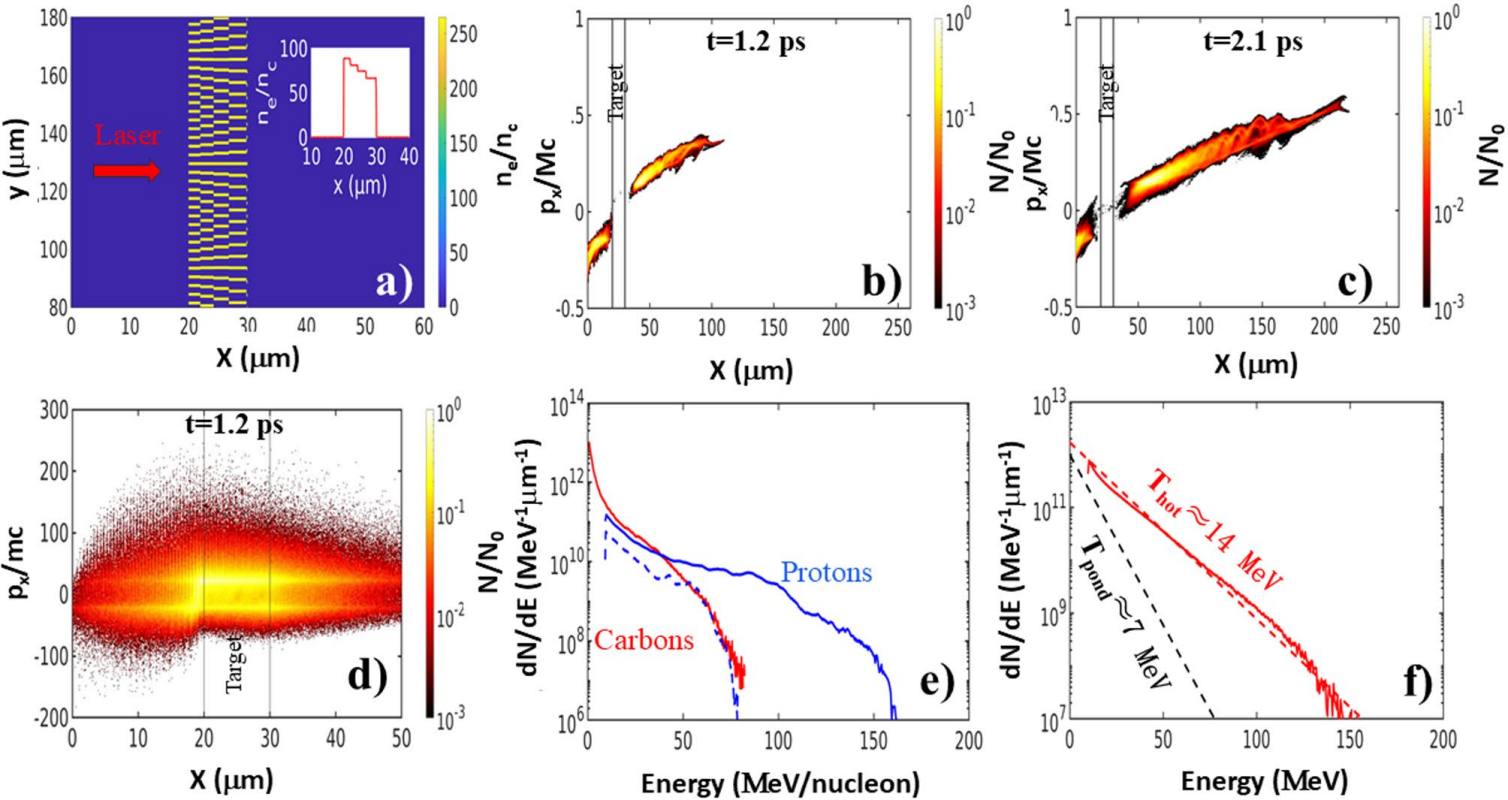
2D OSIRIS modeling of 10 μm LP target interaction with a laser. **(a)** A cross-section of a simplified 10 μm log-pile wire microstructure used for 2D PIC simulations. The CH wire density is 265n_c_. In simulations, the peak laser intensity corresponds to a_0_ = 20. **(b)**-**(c)** Snapshots of the proton phase space showing the target normal sheath accelerated protons originated at the rear surface of the target at 1.2 ps **(b)** and 2.1 ps **(c)**. **(d)** electron phase space distribution at t = 1.2 ps. **(e)** Proton (blue) and carbon ion (red) spectra recorded at t = 1.2 ps (blue dashed line) and t = 2.1 ps (blue solid line). **(f)** Solid red line shows the electron spectrum generated in the forward direction on the laser axis at t = 1.2–1.5 ps. Black dashed line indicates electron temperature T_pond_ anticipated for the ponderomotive force heated particles at a_0_ = 20 laser strength and red dashed line is a fit to the simulated electron energy spectrum.

Then for the LP target found optimal in the experiment, we modeled a 10 μm thick wire structure shown in Fig. 6a which has periodicity changing from 2.5 μm at the laser irradiated front to ~ 4 μm at the rear side of the target. In simulations, parameters of both accelerated proton (Fig. 6b, c,e) and electron (Fig. 6d, f) beams agreed qualitatively with the experimental data only for a microstructured hybrid plasma (inhomogeneous plasma). As shown in phase space distributions in Fig. 6b-c for such microstructured plasma the proton momenta, as characteristic to the TNSA, is monotonically increasing in time. The sheath field at the rear surface persists for at least ~ 2 ps. Proton energy in Fig. 6e reaches at this time above 160 MeV. The higher energies for proton and carbon beams in modelling in comparison with the experiment, are likely associated with limitations of 2D modeling in describing laser-plasma dynamics in an intrinsically 3D morphology of the microstructure. Given this limitation, we focus only on qualitative similarities between the simulation results and the experiment. We also numerically analyzed PW laser-plasma interactions with targets of the different length. For example, similar to the experimental observations, simulations showed that a 5 μm LP target disintegrated too fast preventing efficient TNSA of protons (see data in Fig. 2b). The proton spectra generated in 10,25,50 μm thick microstructures have shown that 10–20 μm thickness is about optimal with the marginal drop of the cut-off energy for longer targets. Although for our wire structures, there is lack of knowledge of how these multidimensional effects can modify the 2D results, multiple PIC studies have suggested a reduction coefficient of two in proton energy^47,52,53^ in a better agreement with the experiment.

In Fig. 6d we present the electron phase space distribution at t = 1.2 ps. Here, the LP structure is not disintegrated yet and it is apparent that the electrons right in front of the target and more importantly inside of the target are bunched at the period of λ/2 characteristic to direct acceleration of electrons in the strong laser field. Contribution of the DLA mechanism in simulations is also supported by observation of clear asymmetry in electron population inside of the target towards the higher energies. In Fig. 6f we present the electron spectrum recorded on the laser axis. Similar to the experiment electron temperature for the LP microstructure is larger than that defined by the ponderomotive force heating of electrons (see the dashed line in Fig. 6f). The peak electron temperature of 14 MeV is achieved at 1.2–1.5 ps and then falls by a factor of two at 2.4 ps. The maximum electron energy at t = 1.5ps approaches 150 MeV in a reasonable agreement with the experiment. Such high electron energy is likely due to DLA in the underdense plasma filling the voids because artificial decreasing the wire density in simulations caused an increase in the electron energy. DLA may explain the higher electron and proton energies measured in the ST microstructures because its random wire geometry provides better coupling of electron trajectories with the electrical field of laser. This complicated multidimensional leaser-plasma microphysics is yet to be fully understood.

## Conclusion

We have experimentally demonstrated that 10–20 μm multilayer 2PP 3D-printed microstructures, composed of both periodic and nonperiodic arrangements of ~ 1-µm diameter polymer wires, provide a robust and relatively laser-contrast-immune platform for efficiently converting a PW-power 1 μm laser beam into a high-energy ion beam. These microstructures enhance laser absorption at the front surface, modulated on the scale of the laser wavelength, which increases ion yield, as well as elevate the temperature of laser-heated electrons within the microstructured plasma, thereby boosting the maximum ion energy.

The main experimental observations are as follows. (1) Generation of higher-energy ≥ 110 MeV proton (80 MeV for LP structures) and ~ 350 MeV carbon ion beams with more than 10X higher net ion yield in comparison with a regular few microns thick CH foil. (2) For the given peak laser intensity of 5 × 10^20^ W/cm^2^ optimal acceleration occurs for structures thicker or equal to 10 μm when the averaged plasma density is close to that determined by the relativistically adjusted critical plasma density. For 3D nanoprinted structures, for different laser conditions this can be easily adjusted by choosing the wire diameter and geometry. (3) Nonperiodic 3D stochastic wire structures exhibit higher energy for emitted electron and ion beams than that from log-pile periodic structures. Optimization of laser-plasma interactions in stochastic microstructures holds the promise to reach very high proton kinetic energies but will require further experimental and numerical studies.

The observed results are *qualitatively* reproduced using 2D PIC simulations of these complex true 3D laser-plasma interactions. For higher laser absorption and electron heating, simulations highlight importance of laser interaction with a hybrid microstructured (wire related) overdense plasma surrounded by a near critical/underdense density electron plasma filling the voids. In such plasmas, the sheath magnitude and its lifetime can be enhanced and ions can be effectively accelerated at the rear surface of the target due to the enhanced TNSA mechanism. However, the precise multidimensional physics inside the target’s micropstructure is yet to be fully understood and will be the subject of our future work. As computing power increases, it will be possible to optimize the design of these targets to give the needed energies and yields for multiple applications.

## Methods

### Details of the experiment

The experiments were performed using the Omega EP laser system in the Laboratory for Laser Energetics at the University of Rochester. The central wavelength of the glass laser is 1053 nm and the p-polarized beam was focused with an f/2 off-axis parabolic mirror to a focal spot shown in the inset in Fig. 1a. It represents a fluence map extracted from a high-resolution wavefront sensing diagnostic^54^. These fluence maps are used to calculate the peak vacuum intensity and peak normalized vector potential, a_0_ for each shot. For a typical shot depicted in Fig. 1a, the driver 600 fs FWHM pulse had on a target 838 TW peak power and a peak intensity in the focused spot 5.2 × 10^20^ W/cm^2^ corresponding to a_0_ ≈ 20. The laser beam was focused at normal incidence to the front surface of the target. The interaction pulse had a nanosecond/picosecond prepulse characterized by the ~ 10^−10^ intensity contrast ratio between this prepulse temporal structure and the main pulse.

This prepulse would be sufficient to create an ablation plasma on the front side of the target’s wire structure earlier during the pulse and to generate the critical density plasma on wires. However, the shock launched at the critical surface by the long-prepulse^42^ would have difficulty in reaching the rear surface because of multilayer structure perpendicular to the shock propagation. Also, a recent paper presents a detailed numerical analysis of the OMEGA EP laser prepulse interaction with a submicron flat foil using a combination of hydrodynamic and two-step kinetic models^55^. They conclude, that a 1–2 μm thick over critical density preplasma step can be generated by the low-intensity prepulse at the front on a ns/ps time scale. When the main pulse arrives, it will interact with this overcritical plasma layer but increasing a_0_ during the laser pulse will result in near critical and underdense laser-plasma interactions due to the relativistic transparency factor. Therefore, the laser prepulse effect on a 10–20 μm thick multilayer structure made of 1 μm wires is likely rather insignificant.

Two independent diagnostics were used for measuring the ion spectra generated in the microstructures in the forward direction. Ions and protons on the laser axis and normal to the target’s rear surface were detected through a 250–300 μm pinhole by a Thomson parabola spectrometer placed approximately 80 cm from the target. In such a spectrometer, ions with different charge-to-mass ratios are dispersed by parallel electric and magnetic fields onto distinct parabola tracs in the plane of the imaging plate detector as shown in Fig. 2a. After the shot, phosphor imaging plates were scanned several times to avoid saturation, recorded and the obtained spectra analyzed. Two species protons and C^6+^ ions dominated in the spectra. Note, that even though the charge-to-mass ratio for C^6+^ and O^8+^ ions are very close to each other, the numbers of O^8+^ were more than two times smaller due to chemical composition of wire material. In order to avoid interference between the protons and carbon ions in the high-energy side, we installed a 1 mm thick Al or W plate-filter to cut-off high-energy ions. In Fig. 2a we show a typical data set of the TP with the Al filter screening above 62 MeV ions recorded both for a 10 μm thick LP structure and a 5 μm thick flat CH foil.

Another proton diagnostic relied on radiochromic films in stack configuration, which provides information on the particle spectrum as well as spatial distribution of the accelerated proton beams. This detector was positioned 5 cm downstream from the laser focus and was designed to have a 15 mm hole in the middle for simultaneous TP measurements on-axis. An overall size of RCFs 60 × 60 mm^2^ at this standoff distance allowed for capturing approximately 60 degrees of the full proton beam divergence. Each RCF stack consisted of up to 13 radiochromic films of different types HD-42 and EBT3, interleaved with metallic foils. By using Al and Ta foils of different thickness, the total detectable energy in the stack was extended to 110 MeV. To increase the spectral resolution beyond a few Bragg peak values corresponding to each RCF, an Al or W multi-step metallic plate was placed in front of the stack. Such proton beam imager and energy spectrometer allowed to have 9 channels in each of 2 × 2 mm^2^ “city block” repeatable structure and, therefore for each shot more than 100 data points could be recovered in the PROBIES energy spectrum^44^. As the result each of the radiochromic films covered the range of energies. The energy resolution of the PROBIES spectrum with an Al step-filter varied in the range of 10 to 110 MeV but was better than a few MeV. For example, Fig. 3a demonstrates how two films from the stack provided spatial distribution of the proton beam for 24–34 MeV and 76–86 MeV energy ranges. Figure 3c presents an example proton beam distribution from a 10 μm LP structure, as measured by the RCF detector covering the energy range of 97–110 MeV as well as lower 76-96 MeV energies.

We also employed regular permanent magnet electron spectrometers to measure spectra of accelerated hot electrons at 27° and 0° with respect to the laser beam axis. For each laser shot, at 25° in the forward direction above the target the X-ray spectrum of hot plasma was recorded and analyzed.

### Fabrication of two-photon polymerization (2PP) 3D laser-printed wire microstructures

Log-pile and stochastic lattice microstructures were produced at General Atomics using in-house built laser scanning 2PP lithography system. To achieve high precision 3D nanofabrication, the system utilizes key components including in sequence a tunable Ti: sapphire femtosecond laser, acousto-optic modulator (AOM), scanning mirror galvanometers (galvos), oil immersion objective lens, and precision XYZ stage system. 2PP polymer precursor is a viscous resin consisting of 40wt% dipentaerythritol penta-/hexa-acrylate (C_55_H_66_O_25_) and 60wt% bisphenol. To this polymer precursor 0.1wt% of highly sensitive two-photon absorbing photo-initiator (4,4’-((1E,1’E)-(2-((2-ethylhexyl)oxy)−5-methoxy-1,4-phenylene)bis(ethene-2,1-diyl))bis(N, N-dibutylaniline)) was added.

During fabrication, a droplet of the 2PP resin is placed on a glass coverslip that is affixed to the precision XYZ stage and then the oil immersion objective lens is dipped into the resin. The substrate interface is located by observing with a camera the appearance and absence of fluorescence during translation of the sample along the optical axis while the laser is being fired. Once the glass coverslip interface is located, fabrication programs are executed that consist of a series of commands to the AOM, galvos, and XYZ stages.

All targets are produced in a printed washer which has a thickness of 60 μm and outer diameter of 300 μm and inner diameter of 200 microns. For log-pile structures, the laser scanner moves linearly along one axis to polymerize a line and then jumps a prescribed hatching distance along the other axis before polymerizing a parallel line. Once a layer is complete, the Z optical axis will translate and polymerized lines will be written perpendicularly to the prior layer. In the resulting log-pile each cross-sectional layer of the log-pile is translationally moved by roughly one micron. Thus after 10 such layers the 11th layer is nearly perfectly overlapped with the first layer. By doing this we ensure that all the laser photons entering the void regions are intercepted by one layer of polymerized line and that there is virtually no transmission of the laser photons beyond this thickness. For gradient density log-pile structures, the hatching distance and thereby the cross-sectional areas of the void is increased as Z optical axis position in increased. Decreasing the plasma density by increasing the void size is expected to homogenize the hot electron density within the sheath region is chosen to vary depending on the Z optical axis position. Multiple wire microstructure surrogates produced with uniform hatching distances were fabricated to gravimetrically characterize bulk densities.

For 3D stochastic lattice structures, a model is produced by generating a random list of points in a volume with the distance between the points equal to the cell size. A Voronoi algorithm is then used to define struts forming cells around the random point list. This technique for modeling lattices was chosen to emulate the random morphology of chemically casted foams while producing a repeatable structure for enhanced deterministic control over the laser-plasma interactions. The stochastic lattice structure is sliced into individual layers along the optical Z axis with each layer consisting of 2D image of points. The galvo moves to each individual point and fires the laser using the AOM to control the exposure time. Once a layer of points is marked, the Z axis is translated and the next layer is fabricated until the whole structure is produced.

After the laser fabrication is completed, the targets are post processed via solvent exchanging of unpolymerized resin in ethanol with multiple rinses. Once any residual resin has been removed, the ethanol is exchanged with liquid carbon dioxide which is then brought to super critical state which is in turn exchanged with air. Samples are characterized with a variety of techniques including bright field microscopy, scanning electron microscopy, and X-ray radiography.

### OSIRIS simulations

Two-dimensional simulations were carried out using the fully kinetic, fully relativistic particle-in-cell code OSIRIS − 4.0 (ref. 49). The simulation box has a size of 260 × 260 *µ*m with a resolution of 63 cells/*λ* in the longitudinal direction and 63 cells/*λ* in the transverse direction, where *λ* stands for the laser wavelength. Electron, proton, and carbon species were modeled considering complete ionization and using the real mass ratio between species. The laser pulse enters the simulation from the vacuum region on the left-hand side of the box and is linearly polarized in the plane of the simulation *y* direction, where *x* is the direction of the laser propagation, with a FWHM pulse duration of 600 fs and a wavelength of 1,053 μm. It was focused at the surface of the LP structure with a spot size of 46 *µ*m (FWHM of the electric field) and a peak *a*_0_ = 20. The LP structure is modeled as an array of preformed plasma wires where both the electrons and ions are allowed to move. The wire plasma density was chosen to be 265 n_c_. In the reference simulation presented in Fig. 5, the total length (thickness) of the LP structure was 10 *µ*m with the spacing between wires varying from 2.5 *µ*m at the front side (laser irradiation side) to 3.5 *µ*m at the rear side. In order to optimize the LP length, we have performed a series of simulations with the same parameters but where we varied the total LP length from 5 to 50 *µ*m. Additionally, to study the importance of the wire structure, we have performed simulations with the same parameters but with a transversely uniform plasma profile with the same average density of the LP structure. All simulations use periodic boundary conditions in the transverse direction and open in the longitudinal direction for both particles and fields. We used 36 particles/cell per species and cubic particle shape for improved numerical accuracy. The time step is dt = dx/sqrt(2), satisfying the Courant condition.

## Data Availability

Data sets generated during the current study are available from the corresponding author on reasonable request.

## References

[1] Hooker, S. M. Developments in laser-driven plasma accelerators. *Nat. Photonics*. **7**, 775–782 (2013).

[2] Macchi, A., Borghesi, M. & Passoni, M. Ion acceleration by superintense laser-plasma interaction. *Rev. Mod. Phys.* **85**, 751–793 (2013).

[3] Borghesi, M. et al. Electric field detection in laser-plasma interaction experiments via the proton imaging technique. *Phys. Plasmas*. **9**, 2214–2220 (2002).

[4] Mackinnon, A. J. et al. Proton radiography of a laser-driven implosion. *Phys. Rev. Lett.* **97**, 045001 (2006). 16907580 10.1103/PhysRevLett.97.045001

[5] Dromey, B. et al. Picosecond metrology of laser-driven proton bursts. *Nat. Commun.* **7**, 10642 (2016). 26861592 10.1038/ncomms10642 PMC4749984

[6] Patel, P. K. et al. Isochoric heating of solid-density matter with an ultrafast proton beam. *Phys. Rev. Lett.* **91**, 125004 (2003). 14525369 10.1103/PhysRevLett.91.125004

[7] Malka, V. et al. Practicability of proton therapy using compact laser systems. *Med. Phys.* **31**, 1587–1592 (2004). 15259663 10.1118/1.1747751

[8] Zeil, K. et al. Dose-controlled irradiation of cancer cells with laser-accelerated proton pulses. *Appl. Phys. B*. **110**, 437–443 (2013).

[9] Kroll, F. et al. Tumour irradiation in mice with a laser-accelerated proton beam. *Nat. Phys.* **18**, 316–322 (2022).

[10] Linz, U. & Alonso, J. What will it take for a laser-driven proton accelerators to be applied to tumor therapy. *Phys. Rev. STAB.* **10**, 094801 (2007).

[11] Vozenin, M. C. et al. Biological benefits of ultra-high dose rate FLASH radiotherapy: sleeping beauty awoken. *Clin. Oncol.* **31**, 407–415 (2019). 10.1016/j.clon.2019.04.001 PMC6850216 31010708

[12] Bourhis, J. et al. Treatment of the first patient by FLASH-radiotherapy. *Radiother Oncol.* **139**, 18–22 (2019). 31303340 10.1016/j.radonc.2019.06.019

[13] Farr, J. et al. Ultra-high dose radiation production and delivery systems intended for FLASH. *Med. Phys.* **49**, 4875–4911 (2022). 35403262 10.1002/mp.15659 PMC9544515

[14] Cowan, T. E. et al. Ultralow emittance, multi-MeV proton beams from a laser virtual-cathode plasma accelerator. *Phys. Rev. Lett.* **92**, 204801 (2004). 15169357 10.1103/PhysRevLett.92.204801

[15] Toncian, T. et al. Ultrafast laser-driven microlens to focus and energy select mega-electron volt protons. *Science* **312**, 410–413 (2011). 10.1126/science.1124412 16484452

[16] Schollmier, M. et al. Controlled transport and focusing of laser-accelerated protons with miniature magnetic devices. *Phys. Rev. Lett.* **101**, 647–653 (2008). 10.1103/PhysRevLett.101.055004 18764401

[17] Noda, A. et al. Phase rotation scheme of laser-produced ions for reduction of the energy spread. *Las Phys.* **16**, 647–653 (2006).

[18] Wilks, S. et al. Energetic proton generation in ultra-intense laser-solid interactions. *Phys. Plasmas*. **8**, 542–549 (2001).

[19] Fuchs, J. et al. Laser-driven proton scaling laws and new paths towards energy increase. *Nat. Phys.* **2**, 48 (2006).

[20] Margarone, D. et al. Laser-driven proton acceleration enhancement by nanostructured foils. *Phys. Rev. Lett.* **109**, 234801 (2012). 23368211 10.1103/PhysRevLett.109.234801

[21] Passoni, M. et al. Toward high-energy laser-driven ion beams: nanostructured double-layer targets. *Phys. Rev. AB*. **19**, 061301 (2016).

[22] Khaghani, D. et al. Enhancing laser-driven proton acceleration by using micro-pillar arrays at high drive energy. *Sci. Rep.* **7**, 44030 (2017). 28900164 10.1038/s41598-017-11589-z PMC5596005

[23] Lubcke, A. Prospects of target nanostructuring for laser proton acceleration. *Sci. Rep.* **7**, 11366 (2017). 28290479 10.1038/srep44030 PMC5349587

[24] Bailly-Grandvaux, M. et al. Ion acceleration from microstructured targets irradiated by high-intensity picosecond laser pulses. *Phys. Rev. E*. **102** (R), 021201 (2020). 32942368 10.1103/PhysRevE.102.021201

[25] Keppler, S. et al. Intensity scaling limitations of laser-driven proton acceleration in the TNSA regime. *Phys. Rev. Res.* **4**, 013065 (2022).

[26] Henig, A. et al. Radiation-Pressure acceleration of ion beams driven by circularly polarized laser pulses. *Phys. Rev. Lett.* **103**, 245003 (2006). 10.1103/PhysRevLett.103.245003 20366205

[27] Alejo, A. et al. Stabilized radiation pressure acceleration and neutron generation in ultrathin deuterated foils. *Phys. Rev. Lett.* **129**, 1144801 (2022). 10.1103/PhysRevLett.129.114801 36154426

[28] Palaniyappan, S. et al. Efficient quasi-monoenergetic ion beams from laser-driven relativistic plasmas. *Nat. Commun.* **6**, 10170 (2015). 26657147 10.1038/ncomms10170 PMC4682178

[29] Wagner, F. et al. Maximum proton energy above 85 MeV from the relativistic interaction of laser pulses with micrometer Thick CH_2_ targets. *Phys. Rev. Lett.* **116**, 205002 (2016). 27258872 10.1103/PhysRevLett.116.205002

[30] Dover, N. P. et al. Enhanced ion acceleration from transparency-driven foils demonstrated at two ultraintense laser facilities. *Light: Sci. Appl.* **12**, 71 (2022). 10.1038/s41377-023-01083-9 PMC10011581 36914618

[31] Higginson, A. et al. Near-100 MeV protons via laser-driven transparency-enhanced hybrid acceleration scheme. *Nat. Commun.* **9**, 724 (2018). 29463872 10.1038/s41467-018-03063-9 PMC5820283

[32] Ziegler, T. et al. Laser-driven high-energy proton beams from cascaded acceleration mechanisms. *Nat. Phys.* **20**, 1211 (2024).

[33] Wan, Y. et al. Physical mechanism of the transverse instability in radiation pressure ion acceleration. *Phys. Rev. Lett.* **117**, 234801 (2016). 27982647 10.1103/PhysRevLett.117.234801

[34] Dorrer, C. et al. OPCPA front end and contrast optimization for the OMEGA EP kilojoule, picosecond laser. *J. Opt.* **17**, 094007 (2015).

[35] Hahn, V. et al. 3D-laser nanoprinting, *Opt. & Photonics News*, October, 28–35, (2019).

[36] Li, M. et al. Low-temperature 3D printing of transparent silica glass microstructures. *Sc Ad*. **9**, eadi2958 (2023). 10.1126/sciadv.adi2958 PMC10550221 37792949

[37] Roberts, E. et al. 3D-patterned inverse-designed mid-infrared metaoptics. *Nat. Commun.* **14**, 2768 (2023). 37179338 10.1038/s41467-023-38258-2 PMC10183040

[38] Luo, H. C. et al. Grayscale two-photon 3D printed gradient-refractive-index metamaterial lens for dual-band mid-infrared imaging. *APL Photonics*. **9**, 051303 (2024).

[39] Dozieres, M. et al. Optimization of laser-nanowire target interaction to increase proton acceleration efficiency. *Plasma Phys. Control Fusion*. **61**, 065016 (2019).

[40] Vallieres, S. et al. Enhanced laser-driven proton acceleration using nanowire targets. *Sci. Rep.* **11**, 2226 (2021). 33500441 10.1038/s41598-020-80392-0 PMC7838319

[41] Ferry, J. et al. Enhancement of laser-driven ion acceleration in non-periodic nanostructured targets. *J. Plasma Phys.* **26**, 905860101 (2020).

[42] Batani, D. et al. Effects of laser prepulses on laser-induced proton generation. *New. J. Phys.* **12**, 045018 (2010).

[43] Pak, A. et al. Collisionless shock acceleration of narrow energy spread ion beams from mixed species plasmas using 1 μm lasers. *Phys. Rev. AB*. **21**, 103401 (2018).

[44] Mariscal, D. A. et al. Design of flexible proton beam imaging energy spectrometers (PROBIES). *Plasma Phys. Control Fusion*. **63**, 114003 (2021).

[45] Rusby, D. R. et al. Effect of Rear surface fields in hot, refluxing and escaping electron populations via numerical simulations. *High-power Laser Sci. Eng.* **7**, e45 (2019).

[46] Kruer, W. L. & Estabrook, K. JxB heating by intense laser light. *Phys. Fluids*. **28**, 430–432 (1985).

[47] Jiang, S. et al. Effects of front-surface target structures on properties of relativistic laser-plasma electrons. *Phys. Rev. E*. **89**, 013106 (2014). 10.1103/PhysRevE.89.013106 24580345

[48] Shaw, J. L. et al. Role of direct laser acceleration in a laser Wakefield electron accelerator with ionization injection. *Phys. Rev. Lett.* **118**, 064801 (2017). 28234524 10.1103/PhysRevLett.118.064801

[49] Pukhov, A. et al. Particle acceleration in relativistic plasma channels. *Phys. Plasmas*. **6**, 2847 (1999).

[50] Willingale, L. et al. High-power, kilojoule laser interactions with near-critical density plasma. *Phys. Plasmas*. **18**, 056706 (2011).

[51] Fonseca, R. A. et al. OSIRIS: a three-dimensional, fully relativistic particle in cell code for modeling plasma-based accelerators, *Lecture Notes in Computer Science*, 342–351, Springer, Berlin (2002).

[52] Fedeli, L. et al. Ultra-intense laser interaction with nanostructured near-critical plasmas. *Sci. Rep.* **8**, 3834 (2018). 29497130 10.1038/s41598-018-22147-6 PMC5832818

[53] Xiao, K. D. et al. Multidimensional effects on proton acceleration using high-power intense laser pulses. *Phys. Plasmas*. **25**, 023103 (2018).

[54] Bromage, J. et al. A focal-spot diagnostic for on-shot characterization of high-energy Petawatt lasers. *Opt. Express*. **16**, 16561–16572 (2008). 18852765

[55] Huang, C. K. et al. Characterization of laser-accelerated proton beams from a 0.5 kJ sub-picosecond laser for radiography applications. *Phys. Plasmas*. **32**, 033107 (2025).

